# mSphere of Influence: The integral art of resolving host-virus interactions

**DOI:** 10.1128/msphere.01040-24

**Published:** 2025-03-20

**Authors:** Elliott D. SoRelle

**Affiliations:** 1 Department of Microbiology and Immunology, University of Michigan Medical School 12266 , Ann Arbor, Michigan, USA; University of Michigan, Ann Arbor, Michigan, USA

**Keywords:** Epstein-Barr virus, viral pathogenesis, host-pathogen interactions, gene regulation, genome organization, genomics, chromatin remodeling, fluorescence microscopy, single-cell sequencing, multiomics, art and science, biotechnology

## Abstract

Elliott D. SoRelle studies viral infection and pathogenesis, specifically Epstein-Barr virus and its associated diseases, through the lens of single cell and spatial biology. In this mSphere of Influence article, he reflects on the essential value of art in biological research—and the frequent homology between the two. He incorporates themes from music and visual arts into a discussion of how three publications entitled “The cybernetics of development” by C. H. Waddington (*The Strategy of the Genes*, chapter 2, https://doi.org/10.4324/9781315765471), “Epstein-Barr viral productive amplification reprograms nuclear architecture, DNA replication, and histone deposition” by Y.-F. Chiu et al. (Cell Host Microbe 14:607–618, 2013, 10.1016/j.chom.2013.11.009), and “Comprehensive integration of single-cell data” by T. Stuart et al. (Cell 177:1888–1902.e21, 2019, https://doi.org/10.1016/j.cell.2019.05.031) shape his scientific perspectives in single-cell virology and provide a conceptual framework for dissecting multifaceted host-virus interactions.

## COMMENTARY

**Fig 1:**
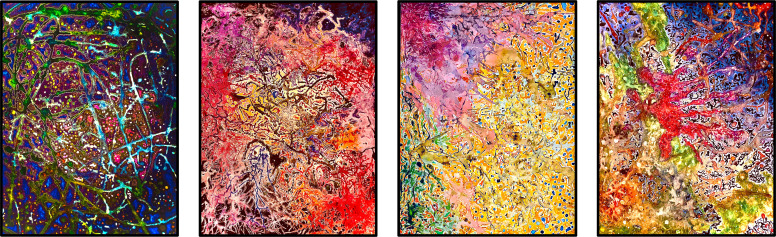
*Die fröhliche Wissenschaft* (2021). Watercolor series painted with p20, p200, and p1000 pipettes. (Copyright Elliott D. SoRelle.)

Conceptual models are no substitute for quality data. Experimental systems are inescapably abstracted from genuine biological articles. Yet, models are critically important in science. Analogies between art and science can likewise be imprecise and fraught. Having made that disclaimer, will you spare some creative license and hold my metaphorical beer? I humbly ask because art is inextricable from my broader sense of science, biological complexity, and technology’s specific role in comprehending the nature and effects of host-virus interactions. Here, I offer admittedly subjective thoughts on three films and their relations to scientific visualizations I find vital in my new lab. For different reasons, each one left me wide-eyed and slack-jawed in awe upon first viewing; they continue to inspire me in the possibility of “Die fröhliche Wissenschaft” (1) (Fig. 1).

                                                                     ❚

Kubrick and Clarke’s *2001: A Space Odyssey* (2) is elegantly timeless. Renowned for cinematographic innovation and explorations of humanity vis-à-vis technology, it is urtext for other science-fiction classics, a perennial source for parody, and a cultural influence some 56 years on. *2001*’s artistic success and resilience are ingrained in its structure, which honors older works inspired by even older philosophies. Nominally three acts (“The Dawn of Man,” “The Jupiter Mission,” and “Jupiter and Beyond the Infinite”), the “and” in the third title implies four key movements (as does the fourfold appearance of the film’s memorable touchstone). This structure mimics musical leitmotifs in the first movement of Strauss’s instantly recognizable tone poem *Also sprach Zarathustra* (*Sonnenaufgang*, or “Sunrise”) (3), which was in turn inspired by Nietzsche’s eponymous four-volume novel (4) and bookends *2001*. It is fitting, then, that the concept of eternal return (recurrence) (5) subtends each work. Rendered in contemporary media, these works iteratively meditate on a model of existence, each elaborating and refining its underlying substance. This formula—rhyming with history, systematically re-examining old models with new tools, updating priors—is an essential kernel in the pursuit of an understanding progressively less incorrect. *2001* artistically reflects advancement by scientific method, synthesizing a foundational cinematic model. Chapter 2 of Waddington’s *The Strategy of the Genes* (6) shares structural homology with *2001*. Working from first principles, Waddington applies mathematical and physical reasoning to formulate a framework for the emergence of phenotypically discontinuous cells from a common progenitor. Figures 1, 3, 4, and 5 in chapter 2 (6) are a visual tour de force in the Straussian poetic tradition. First, the rudiments: a gene network diagram evoking hierarchical regulation of phenotype. This concept is propagated into phase space depicting discrete cell fates derived from initially continuous phenotypic space. The visual triumph of chapter 2’s famous epigenetic landscape is the translation of these mathematical concepts into an easily ascertained physical analogy for cell fate, akin to *Sonnenaufgang’s* “aha” after the leitmotif’s third invocation. Figure 5 is the resplendent fanfare, wherein Waddington brings readers full circle by revealing gene network activity undergirding cell fate. It is a generalization—a theory from rough observations and reasoning without mechanistic evidence. However, this model was remarkably advanced in its time (the DNA double-helix structure was published only four years prior [7]) and endures as a potent visual expression of epigenetically governed identity. It is emblematic of artistic abstraction operating as a scientific lodestar—a shared reference from which we might understand and dissect phenomena more perfectly.

                                                                    ❚

What might we learn from extrapolating Waddington’s concept beyond development? If coherent cellular identity is founded in the epigenomic architecture of DNA methylation, histone modifications, chromatin looping, and higher-order compartmentalization, then intranuclear viruses make things a hell of a lot messier. That’s my unscientific conclusion from the strangely beautiful fluorescence microscopy of Epstein-Barr virus (EBV) latent-to-lytic reactivation in Chiu et al. (8) (and recently, Rosemarie et al. [9]). Using a fluorescent reporter virus, Chiu et al. visualize concomitant formation of nuclear viral replication compartments, chromatin reorganization, histone relocation, and downregulation of histone chaperones. Figure 4A and C and especially Fig. 5B in Chiu et al. are vivid depictions of collateral disorder inflicted on the cellular genome during EBV reactivation. Rosemarie et al. further identify viral elements necessary to induce reorganization of cellular chromatin (ROCC)—the immediate early genes *BZLF1* and *BRLF1*, the origin of lytic replication, and DNA synthesis genes *BALF5*, *BALF2*, *BBLF2/3*, *BBLF4*, *BSLF1*, *BMLF1*, and *BMRF1* (9). They define two distinct ROCC flavors; both require *expression* of viral DNA synthesis genes, but only one (ROCC type II) requires viral DNA synthesis. Of course, many lytic cells (particularly ROCC type II) incur irreparable damage and die. The final behaviors of such doomed cells may be relevant to pathogenesis, though any impact of sustained epigenetic alterations would be moot. What about epigenomic changes in cells with incomplete EBV reactivation (i.e., ROCC type I)? I suspect ROCC types I and II are but two points in the EBV reactivation fate landscape—“each unhappy family is unhappy in its own way” (10). As in Kwan and Scheinert’s masterful *Everything Everywhere All at Once* (11), even seemingly quotidian relationships (parent-child, or host-virus) are multidimensionally layered with complexity (think of how many things are happening simultaneously in our model systems versus how few parameters we typically assay). *Everything* thrives in its characters’ evolving inter-relational dynamics cast into visual mutations and transitions through worlds within worlds. The film’s sonically stunning counterpart, *This is a Life* performed by Son Lux, Mitski Miyawaki, and David Byrne, is particularly evocative of the extreme heterogeneity and complexity within interwoven entities: “Many lives that could have been…entangled for eternity” (12). This is the same sound and fury (13) of viral and host genomes in conversation, so to speak, across cell populations. Having seen the extent to which EBV and other DNA viruses physically upend genomic chromatin compartmentalization (to say nothing of alterations to host gene regulation mediated by virally-encoded transcription factors [14–16], RNA binding proteins [17], and other mechanisms [18]), into what mysterious parallel universe(s) of genomic dysregulation might individual cells voyage if they remain viable after viral reactivation commences? In which panel of *The Garden of Earthly Delights* (19) do such cells reside? In light of the intriguing but incompletely resolved involvement of EBV reactivation in tumorigenesis (20–22) (and cellular dysregulation during viral reactivation more generally), might some of these interspecies genomic conversations—those signifying more than nothing—be biologically pertinent to disease? How do we define and dissect them to obtain the “view of other worlds from our window sills?” (12).

                                                                    ❚

The answer necessarily resides in single-cell measurements. In a recent scRNA-seq study of EBV lytic reactivation, my colleagues and I identified heterogeneous fates including the emergence of *BZLF1*^+^ cells expressing genes associated with developmental pluripotency and self-renewal (23). In certain respects, this finding echoes responses observed in cells infected with HSV-1 (24) as well as reactivation-induced expression of embryonic stage pioneer factors conserved across multiple DNA viruses (25). However, the mere technology of scRNA-seq is inadequate for comprehending the fate landscape of viral reactivation. It is ultimately an assay to be applied to well-posed biological questions and tractable experimental perturbations. Moreover, scRNA-seq captures only one level in the regulatory hierarchy spanning genome to cellular function. What epigenomic regulatory states, microscopic phenotypes, and biological responses correspond to scRNA-seq clusters? Conceptually, we might start making sense of how to address such questions of interrelation by distilling Demme’s *Stop Making Sense* (26), a master class in storytelling constructed from performances by Talking Heads. The logic and progressive layering of the cinematography and concert setlist stand out. The film begins with a tracking shot of David Byrne walking onto a stage with no set elements or backdrop. The concert begins with a backing beat and Byrne’s acoustic performance of “Psycho Killer.” Tina Weymouth joins for “Heaven,” and then song after song, each additional band member joins the stage—Chris Frantz on drums, Jerry Harrison on guitar, Steve Scales on percussion, Lynn Mabry and Ednah Holt on backup vocals, Alex Weir on guitar, and Bernie Worrell on keys. Set elements are added along the way, the energy crescendos, and the band proceeds to burn down the house. They evolve throughout the show. They switch instruments. Members come and go—they transform into a different band (Tom Tom Club). Byrne disappears completely for Tom Tom Club’s “Genius of Love” and returns in the Big Suit, reinvented. The experience is one of witnessing elemental construction with little artifice; the sound and stage crewmembers are critical characters in the story, not people to be concealed or forgotten. The bandmembers’ talents are complementary, and their instruments—the molecular layers—build an increasingly comprehensive, unified sound. For the team and the music, the sums are greater than the constituent parts. Each song (cell) should be understood by more than a single instrument’s representation. In single-cell biology, a powerful visualization of such multimodal layer unification resides in Fig. 1C, 3A, and 5A in Stuart et al.’s landmark publication of Seurat v3 (27) (named for the French Post-Impressionist, a nod to the pointillist style of dimensionally reduced cell plots). The authors develop computational methods to identify “anchor” features to join information from different levels (expression, genome accessibility, spatial organization, and—conceivably—non-sequencing techniques) at cellular resolution. This is a remarkably powerful capability—as illuminating as its application allows. Pushing this type of integrative analysis in the context of mechanistic experiments will revolutionize host-virus interaction studies. Waddington could not have imagined the possibilities in his wildest fever dream.

                                                                ❚

So, we iterate. In complementary, multidisciplinary teams, we eternally return to our models again and again. Technological advances make it worthwhile to revisit “old” yet staggeringly multifaceted biology with new faculties and fresh perspectives, so that we might discover things that have been there all along. We should be careful not to overemploy technologies for their own sake (colloquially, “driving a Ferrari to the grocery store”), and certainly we should not over-delegate matters of critical thinking to artificial intelligence (arguably, *2001*’s most enduring lesson). This goes doubly in the knotty realm of generative AI. These are powerful and versatile tools that draw on an expanse of human knowledge and content, but dig deep enough into a chatbot “conversation” and you’re apt as not to end up in an infinite, substance-less loop. Ever again, a tool is as good or bad as its use. By contrast, art can and should be more. Engaging with it can evoke, instruct, and even function as a whetstone for analytical scrutiny. Art’s perception can be a powerful heuristic for hypothesis generation and creative experimental design. We should envision a rigorously practiced science that remains keen to and invigorated with the formal syntax of artistic expression, a primordial yet ever-adapting spring of humanity. If we do, “Watch out—you might get what you’re after…” (26).

### Spheres of influence audio

Music referenced herein (plus a fourth “movement”) is available as an accompanying playlist: spheres_of_influence.
